# Strengthening Respiratory Virus Surveillance in Sub‐Saharan Africa: Integrated Epidemiological and Genomic Monitoring in Côte d'Ivoire

**DOI:** 10.1111/irv.70239

**Published:** 2026-02-18

**Authors:** Hervé A. Kadjo, Daouda Coulibaly, Yakoura Karidja Ouattara, Sylla Aboubacar, Diané Maxime, Nguessan Konan, Kouakou Luc‐Venance, Kouassi Helene, Mboua Jean Marc, Kouakou Bertin, Adagba Marius, Edgard Adjogoua, Ekra Daniel, Meite Syndou

**Affiliations:** ^1^ Department of Epidemic Viruses Pasteur Institute of Côte d'Ivoire Abidjan Abidjan Côte d'Ivoire; ^2^ Epidemiological Surveillance Department National Institute of Public Hygiene Abidjan Côte d'Ivoire; ^3^ Molecular Biology Platform Pasteur Institute of Côte d'Ivoire Abidjan Abidjan Côte d'Ivoire; ^4^ Biological Resources Center Pasteur Institute of Côte d'Ivoire Abidjan Côte d'Ivoire; ^5^ Bioinformatics Laboratory Pelefero Gon Coulibaly University of Korhogo Korhogo Region des Savanes Côte d'Ivoire; ^6^ Biology Laboratory University Teaching Hospital (CHU) Abidjan Yopougon Côte d'Ivoire; ^7^ Center for the Study of High‐Risk Infectious Pathogens Pasteur Institute of Côte d'Ivoire Abidjan Côte d'Ivoire

**Keywords:** Côte d'Ivoire, epidemiology, genomic, influenza, integrated surveillance, RSV, SARS‐CoV‐2

## Abstract

**Background:**

Following WHO recommendations issued in 2019 and 2022, the National Influenza Center (NIC) of Côte d'Ivoire initiated the integration of Respiratory Syncytial Virus (RSV) and SARS‐CoV‐2 surveillance into its existing sentinel influenza surveillance to strengthen the monitoring of respiratory viruses.

**Methods:**

The national influenza sentinel surveillance protocol was revised to include specific requirement of RSV and SARS‐CoV‐2. Nasopharyngeal swabs were collected between January 2022 and December 2023 for all three viruses and were tested by real time RT‐PCR. Only PCR‐positive samples with Ct value < 28 and adequate sample volume were selected for sequencing. CDC Flu SC2 multiplex rRT‐PCR assay and Oxford Nanopore MinION Mk1C were used; influenza sequencing was performed at CDC Atlanta. Phylogenetic analyses were conducted to identify genotypes, lineages, and assess genetic relatedness to global strains.

**Results:**

Between January 2022 and December 2023, 8316 samples were tested; 12.6% (*n* = 1044) were positive for at least one of the three viruses. RSV (5.63%) detection in severe acute respiratory infection (SARI) cases increased significantly from 3.4% in 2022 to 8.4% in 2023 (*p* < 0.0001). Similarly, influenza (3.71%) detection in SARI cases rose from 1.3% to 2.6% (*p* = 0.0057). SARS‐CoV‐2 (3.22%) detection was significantly associated with age (*p* = 0.002). All three viruses circulated year‐round with distinct seasonal peaks. Genomic analysis showed that A(H3N2) viruses belonged to clade 3C.2a1b.2a.2, A(H1N1) pdm09 to clade 6B.1A.5a.2 and B/Victoria to clade V1A.3a.2, all aligning with global trends. Among SARS‐CoV‐2 cases, BA.2 and BA.5 sublineages of Omicron predominated in 2022 while XBB and XBB.1.5 sublineages emerged in 2023. Whole genome sequencing revealed RSV A strains as genotype A.D.5.1 and RSV B as genotype B.D.E.1.

**Conclusion:**

Integration of RSV and SARS‐CoV‐2 into influenza sentinel surveillance has enabled continuous detection and genomic characterization, reinforcing the critical role of integrated sentinel surveillance for timely response to respiratory virus threats.

## 1 Introduction

Acute respiratory infections (ARIs) are one of the leading causes of death worldwide, particularly in sub‐Saharan African countries [1]. Several studies have demonstrated the major role of viruses in the etiology of ARI [2]. Among these viruses, influenza viruses, RSVs, and, more recently, SARS‐CoV‐2 are the subject of increased sentinel surveillance [3]. These viruses are responsible for annual seasonal epidemics with pandemic potential and significant morbidity and mortality, particularly among children under 5 [4, 5]. Indeed, each year influenza causes, according to the World Health Organization (WHO), around a billion cases of seasonal influenza, including 3–5 million cases of severe illness. It causes 290,000–650,000 respiratory deaths annually. Ninety‐nine percent of deaths in children under 5 years of age with influenza‐related lower respiratory tract infections are in developing countries. Respiratory syncytial virus (RSV) infection has been one of the most common causes of lower respiratory tract infections in infants and young children. The global incidence of RSV‐associated lower respiratory tract infections is estimated at over 30 million cases in children under the age of five, resulting in 3.2 million hospitalizations [6]. The 2020 COVID‐19 pandemic demonstrated the scientific community's interest in monitoring SARS‐CoV‐2. Between 2020 and 2023, SARS‐CoV‐2 caused over 7 million deaths worldwide, with the emergence of several variants. SARS‐CoV‐2, like other viruses, mutates over time. Monitoring these mutations helps in identifying new variants that may spread more easily, cause more severe illness, or evade immunity from vaccines or previous infections. Some variants might reduce the effectiveness of existing vaccines. Continuous monitoring ensures that vaccines can be updated or new ones developed to protect against these variants. Historically, influenza virus surveillance began at the global level in 1952 with the establishment of a sentinel surveillance network that has evolved over the years to become the GISRS [7]. WHO guidelines adopted in 2019 and 2022 to update the protocol of the national surveillance system for the integration of RSV and SARS‐CoV‐2 sentinel surveillance into the existing influenza surveillance network [8, 9]. WHO strategy and guidelines adopted to redefine the national influenza surveillance system in Côte d'Ivoire. Since 2006, Côte d'Ivoire has implemented a sentinel influenza surveillance network. During the period 2022–2023, systematic detection of influenza viruses, RSV, and SARS‐CoV‐2 was carried out the analysis on respiratory samples collected from sentinel sites. This article aims to present the process of integrating RSV and SARS‐CoV‐2 sentinel surveillance into the existing influenza sentinel surveillance network and the results obtained.

## 2 Materials and Methods

### 2.1 Study Design and Period

A descriptive cross‐sectional study was conducted from January 2022 to December 2023 at the National Reference Centre for Influenza and Respiratory Viruses located at the Institut Pasteur in Côte d'Ivoire on the Adiopodoumé site.

### 2.2 Influenza Sentinel Surveillance Network

Cote d'Ivoire started an influenza sentinel surveillance program, part of the Global Influenza Surveillance and Response System (GISRS) in 2006. The network has grown to 10 sentinel sites, five in Abidjan and five in other regions across Côte d'Ivoire. Patients of all ages with severe acute respiratory infection (SARI) and influenza‐like illness (ILI) are enrolled, nasopharyngeal samples taken, and samples sent to the Institute Pasteur of Côte d'Ivoire laboratory in Abidjan for molecular analysis.

### 2.3 Revising Influenza Sentinel Surveillance Protocol

Sentinel surveillance of RSV and SARS‐CoV‐2 through the sentinel influenza surveillance network in Côte d'Ivoire required a revision of our initial national influenza surveillance protocol. The protocol revision was based on WHO guidance documents for integrating RSV and SARS‐CoV‐2 into influenza sentinel surveillance [9]. The following points, objectives, case definition, and notification form have been revised. Monitoring epidemiological trends; evolution of early detection of epidemics; determination of severity factors of influenza, COVID‐19, and RSV infection; descriptive epidemiology associated with ILI or SARI cases; and the monitoring of locally circulating virus types/subtypes or lineages/sublineages and their relationship to global and regional patterns were retained as the main objectives. Concerning case definition, since January 2015, for influenza, the case definition used included ILI, which is an acute respiratory infection with a measured fever of ≥ 38°C and cough occurring within the last 10 days, and SARI was suspected in patients with a historic of fever or a fever ≥ 38°C, a cough with onset of symptoms ≤ 10 days and whose condition required hospitalization. The adaptation of the case definition for influenza/RSV surveillance focused on fever. Fever or the notion of fever was one of the main criteria in defining cases for influenza surveillance. In RSV infection, a significant fraction (often > 50%) of young children and elderly patients infected with RSV present without fever [10, 11]. Therefore, fever or the notion of fever was no longer an absolute criterion for the recruitment of patients. [8]. WHO interim guidance on the integration of COVID‐19 into influenza surveillance revealed that the most common clinical features of COVID‐19 were fever (83%) and cough (60%), followed by loss of taste/smell (41%), fatigue (31%), and loss of appetite (30%) [12]. We added to influenza/VRS case definition “any person with anosmia (loss of smell) or agueusia (loss of taste).” The notification form was modified to take into account the criteria for recruiting suspected cases in accordance with the new case definition, vaccination status (against COVID‐19), and history of COVID‐19.

### 2.4 Samples Collection

**FIGURE 1:**
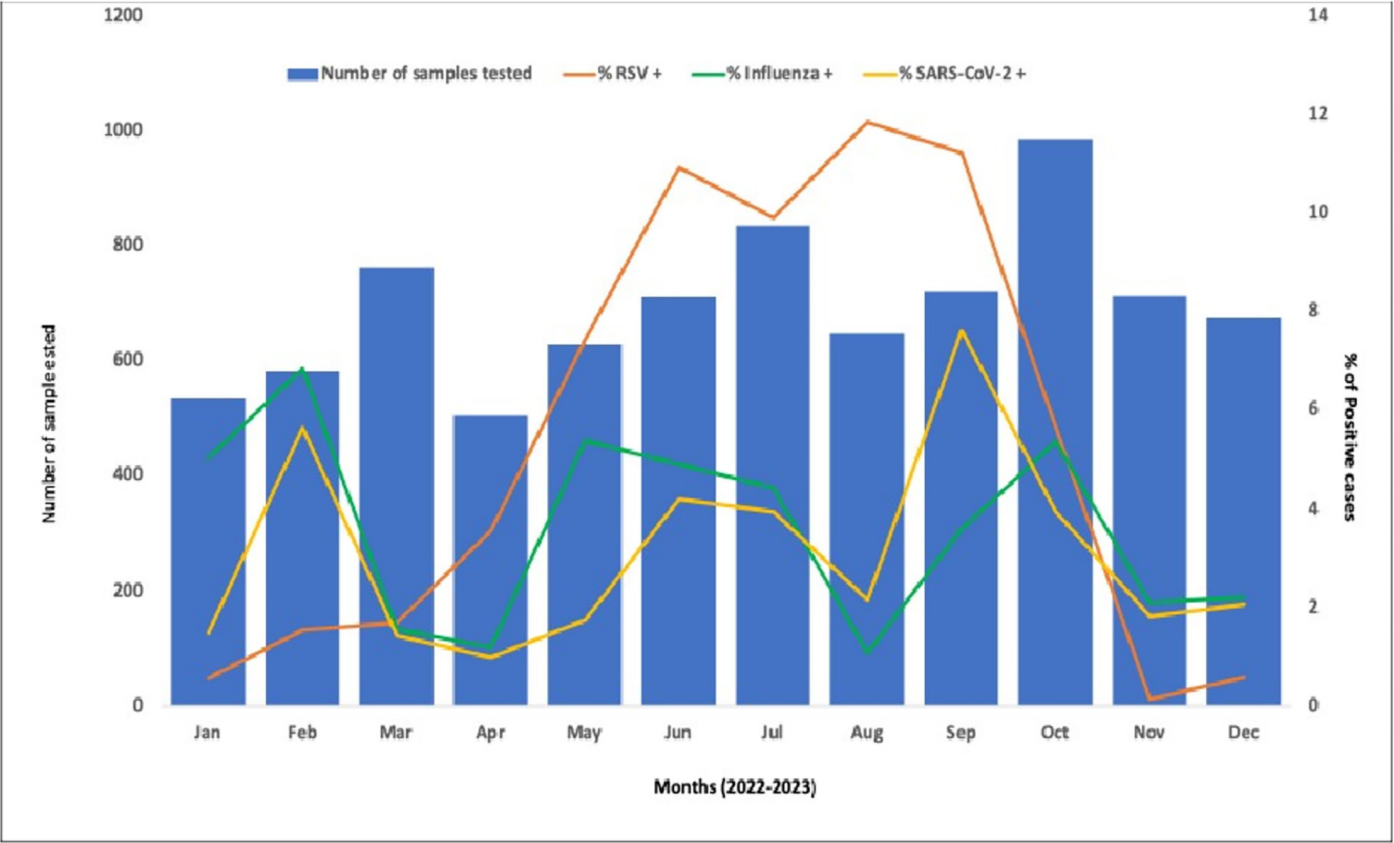
Monthly distribution of laboratory confirmatory cases of RS, Influenza virus and SARS‐CoV‐2 in Cote d'Ivoire, 2022–2023.

Samples were collected between January 2022 and December 2023 as part of the influenza sentinel surveillance system (Figure 1). A nasopharyngeal specimen was collected using Universal Transport Media (UTM3LR) from COPAN. Specimens were kept at +4°C following collection and during transport. Respiratory specimens were transported three times per week (Monday, Wednesday, and Friday) from sentinel sites to the National Influenza Center (NIC) located at the Pasteur Institute. For each ILI and SARI case, a case‐based surveillance form including epidemiological and clinical data such as date of illness onset, date of sample collection, the patient's name, gender, age, medical history, clinical symptoms, and vaccination status were recorded at the time of sample collection.

### 2.5 Molecular Detection

Viral RNA was extracted using the QIAamp Viral RNA Mini Kit (Qiagen, Hilden, Germany) according to the supplier's instructions. For influenza and SARS‐CoV‐2, samples were tested using the CDC influenza SARS‐CoV‐2 (Flu SC2) multiplex real‐time reverse transcription polymerase chain reaction (rRT‐PCR) (CDC Influenza Virus Real‐Time RT‐PCR Influenza A/B Typing Panel (VER 2) (RUO) (Catalog No. FluRUO‐14) as per CDC protocol [13]. All samples were tested for human RNAse P (RP) gene as a housekeeping gene. A second CDC multiplex rRT‐PCR was performed for all influenza A positives to subtype for influenza A(H1N1)pdm09 and AH3 using the CDC Influenza Virus Real‐Time RT‐PCR Influenza A (H3/H1pdm09) Subtyping Panel (VER 3) (RUO) (Catalog No. FluRUO‐15), whereas influenza B positives were genotyped for influenza B Victoria and Yamagata using the CDC Influenza B Lineage Genotyping Panel (RUO) (Catalog No. FluRUO‐11). For RSV, the Matrix protein was the target of the tests carried with the following primers and Probe; forward primer: GCAAATATGGAAACATACGTGAACA; Reverse primer: GCACCCATATTGTWAGTGATGCA; Probe:(ROX) CTTCACGAAGGCTCCACATACACAGCWG‐(BHQ‐2). The Superscript III Platinum One‐step qRT‐PCR Kit was used for amplifications. The Master mix volume of 22.5 μL consisted of 12.5 μL 2X Buffer, 1 μL MgSO4 (2 mM) and 0.6 μL RT/Taq Platinum. Primers and probes (Table) at 0.2 μM were used. A volume of 2.5 μL RNA was added to each identified well. The amplification program was carried out under the following conditions with reverse transcription at 50°C for 30 min, with activation of the enzyme at 95°C for 02 min. The PCR was carried out in 40 cycles with 95 lasting 15 s and 55°C in 30 s.

### 2.6 Sequencing and Bioinformatics Analysis

#### 2.6.1 Influenza

A batch of positive samples was sent to CDC Atlanta for sequencing. All sequences obtained were deposited in the Epi‐FLu database on GISAID. A phylogenetic analysis was performed to determine the relationships between influenza viruses circulating in Côte d'Ivoire with vaccine strains and influenza viruses circulating in other regions. Phylogenetic analysis was constructed with maximum. Like hood (ML)and bootstrap analysis of 1000 replicates using the GTR=G model implemented in MEGA 7. The full‐length HA sequences were used to characterize A(H3N2), A(H1N1) pdm09, and B Victoria lineage strains into genetic clades according to CDC guidelines.

#### 2.6.2 SARS‐CoV‐2

For SARS‐CoV‐2, PCR‐positive samples with low CT value (CT < 28) were selected for whole‐genome sequencing. Sequencing was performed using Oxford Nanopore technology (ONT) with the MinIon MK 1C. ONT library was prepared according to the ARTIC Midnight PCR tiling protocol of the SARS‐CoV‐2 virus with the rapid coding kit (SQK‐RBK110.96) [14]. For sequencing, the SQKRBK110.96 and EXP‐MRT001 kit was used for the preparation of the Priming Mix and the Library Mix which were subsequently loaded onto a Flow‐cell inserted into the MinION Mk‐1C device according to the protocol instructions. When the Run is completed the FastQ‐pass file is retrieved for sequence analysis. Sequence analysis was performed using the ARTIC‐nCoV‐bioinformatics SOP‐v1.1.3 protocol and base calling was performed using Guppy v.4.2.2 (Oxford Nanopore Technologies) in high mode. Precision (model dna_r941_450bps_hac). ARTIC ONT sequencing data were demultiplexed using guppy_barcoder (v4.2.2) with the “require_barcodes_both_ends” option and a score of 60 on both ends. Analysis was performed using a copy of the ARTIC pipeline (v1.1.3) to generate a consensus sequence for each sample in FASTA format. The pipeline includes the following main steps: the input reads were filtered based on read length (ARTIC, 400–700) and mapped to the Wuhan‐Hu‐1 reference genome (Accession MN908947.3) at minimap2 help (Version 2.17‐r941). The trimmed reads were then used for variant calling with medaka (v 1.2.0). The final consensus was generated from a filtered VCF file and a position mask file with a coverage depth less than 20. The quality assessment of SARS‐CoV‐2 genomes, including genomic alignment, clade assignment, Pango lineage designation, and annotation of genetic variations was performed and Phylogenetic tree reconstructed using Next clade (Version 2.9.1.16).

#### 2.6.3 RSV

Sequencing was performed at the WHO Collaborating Centre for Influenza Reference and Research Victorian Infectious Diseases Reference Laboratory the Peter Doherty Institute for Infection and Immunity Melbourne, Australia, using the one‐step RT‐PCR amplification of respiratory syncytial virus (RSV) in two primer pools for whole‐ genome sequencing (WGS) protocol. Phylogenetic analysis was performed on sequenced samples to characterize any temporal and geographical clustering. Firstly, the CDC IRMA pipeline (wonder.cdc.gov/amd/flu/irma, github.com/ammaraziz/wfi) was used for genome assembly and creation of consensus sequence. Secondly, the consensus sequences were aligned to a high‐quality RSV reference alongside publicly available viruses from GISAID and GenBank (www.ncbi.nlm.nih.gov). Thirdly, phylogeny was constructed using the Nextstrain Augur pipeline (github.com/nextstrain/augur).

### 2.7 Statistical Analysis

Epidemiological and clinical characteristics were described using proportions, and group comparisons were assessed through chi‐square test or Fisher's exact test. A *p* value of less than 0.05 was considered statistically significant. The seasonality of RSV, influenza, and SARS‐CoV‐2 circulation was studied by analyzing the monthly distribution of incident positive cases and the proportion of positives over the two study years. This monthly positivity was estimated by dividing positive cases by the total number of suspected cases. The statistical analysis was performed using R Studio Version 4.3.2.

## 3 Results

Between January 2022 and December 2023, a total of 8317 samples were collected through National influenza sentinel surveillance network and tested by the National Influenza Reference laboratory. Of 8317, 5109 (61.4%) were ILI cases and 3207 (38.6%) were SARI cases. The age of population enrolled ranges from 01 months to 101 years. The sex ratio (male/female) was 1/1.9. Children aged 0–2 years represent 42.3% of patients tested and were the most represented in both SARI and ILI cases (*p* < 0.001). Average time between onset of symptoms and sample collection date was 3.2 days. Fever and cough were present in 72.2% and 98.4% of cases. Anosmia and ageusia were found in 2.6% and 1.1% of patients. Molecular testing revealed that 1044 (12.6%) samples were positive for at least one of the three targeted respiratory viruses (influenza viruses, SARS‐COV‐2, RSV). Individually, the positivity rate for influenza was 3.7% (309/8316), 3.2% (268/8316) for SARS‐CoV‐2, and 5.6% (468/8316) for RSV. The three target viruses were detected in both SARI and ILI cases. The most common virus detected in SARI cases was RSV (5.8%), followed by SARS‐CoV‐2 (2.0%) and influenza viruses (1.9%). RSV detection increased significantly between 2022 and 2023, from 3.4% in 2022 to 8.2% in 2023 (*p* < 0.0001). Detection of influenza viruses also increased significantly in SARI cases between 2022 (1.3%) and 2023 (2.6%) (*p* = 0.0057). On the other hand, the overall prevalence of influenza was higher in ILI cases than in SARI cases (4.8% vs. 1.9%). This prevalence increased significantly between 2022 and 2023 (*p* = 0.0068). RSV detection also increased in ILI cases over the 2 years, rising from 3.9% in 2022 to 6.90% in 2023 (*p* < 0.0001). Regarding SARS‐CoV‐2, detection in ILI remained stable over the 2 years, in contrast to SARI, where detection fell slightly without reaching a significant level (*p* = 0.0618) (Table 1).

**TABLE 1:** Distribution of influenza, SARS‐CoV‐2, and RSV cases by years and clinical status.

	Years	2022	2023	Total	*p*
		*n* (%)	*n* (%)	*n* (%)	
	Total of samples collected	3998 (48)	4318 (52)	8316 (100)	
	SARI cases	1657 (41.4)	1550 (35.9)	3207 (38.6)	< 0.0001
	ILI cases	2341 (58.6)	2768 (64.1)	5109 (61.4)	< 0.0001
Virus detected in SARI cases	**Influenza** ^a^	**22 (1.3)**	**41 (2.6)**	**62 (1.9)**	0.0057
	*A(H1N1)pdm09*	1 (4.54)	24 (57.5)	24 (38.71)	
	*H3*	11 (50)	6 (15)	17 (27.4)	
	*B VIC*	10 (45.5)	11 (27.5)	21 (33.9)	
	*B YAM*	0 (0.0)	0 (0.0)	0 (0.0)	
	**SARSCOV‐2** ^a^	**39 (2.4)**	**25 (1.6)**	**64 (2.0)**	**0.0618**
	**RSV**	**56 (3.2)**	**130 (8.2)**	**186 (5.8)**	< 0.0001
Virus detected in ILI cases	**Influenza** ^a^	**94 (4.0)**	**152 (5.49)**	**246 (4.8)**	0.0068
	*A(H1N1)pdm09*	0 (0.0)	78 (0.0)	94 (26.3)	
	*H3*	45 (47.9)	41 (47.9)	151 (42.2)	
	*B VIC*	49 (52.1)	33 (52.1)	113 (31.6)	
	*B YAM*	0 (0.0)	0 (0.0)	0 (0.0)	
	**SARSCOV‐2** ^a^	**86 (3.7)**	**116 (4.2)**	**202 (3.9)**	**0.2225**
	**RSV**	**93 (3.9)**	**191 (6.9)**	**284 (5.6)**	< 0.0001

^a^ Data used for Pearson's chi‐squared test; clinical status = SARI or ILI classification.

Analysis of the distribution of positive cases by age revealed distinct profiles for the three viruses studied. The predominance of influenza cases was reported in children aged 0–2 years, with 25 cases of SARI and 75 cases of ILI, and in adults aged 25–49 years, with nine cases of SARI and 56 cases of ILI. No statistically significant difference was observed between the different age groups and clinical status (*p* = 0.391). With regard to SARS‐CoV‐2 infections, cases of SARI occurred in children aged 0–2 years (33 cases) and in people over 50 years of age (11 cases). The same was observed for SARS‐CoV‐2 infection in the 0–2 (77 cases) and 13–24 age groups. The chi‐square test revealed a significant association between age and SARS‐CoV‐2 (χ^2^ = 18.71, *p* = 0.002), suggesting an association between age and the clinical severity of SARS‐CoV‐2 infection. With regard to RSVs, the majority of RSV positive cases were observed in children in the 0–2 age bracket (137 cases of SARI and 208 cases of ILI) (Table 2).

**TABLE 2:** distribution of influenza, SARS‐CoV‐2 and RSV according age group and clinical status (SARI, ILI), Cote d'Ivoire, 2022–2023.

Age group (years)	Influenza (SARI)	Influenza (ILI)	SARS‐CoV‐2 (SARI)	SARS‐CoV‐2 (ILI)	RSV (SARI)	RSV (ILI)
0–2	25	75	33	77	137	208
3–5	13	36	5	13	16	21
6–12	5	25	4	15	2	5
13–24	1	32	4	26	3	10
25–49	9	56	7	56	10	21
> 50	9	21	11	15	18	19
**Total**	**62**	**245**	**64**	**202**	**186**	**284**

*Note:* RSV (χ^2^ = 78.53, *p* ≤ 0.0001).

The monthly distribution of positive cases revealed a distinct seasonal profile for the three targeted viruses. Two circulation peaks were observed with the influenza viruses: a first peak in February, followed by a second, more significant peak in October. SARS‐CoV‐2 circulated throughout the year with two distinct peaks. The first of which overlapped with that of influenza viruses in February, and the second in September. As for the RSV, circulation was marked between the months of May and October, with a peak in incidence in July. Detection levels for all three viruses were lowest in April. The months of November and December were characterized by a drop in virus circulation, particularly RSV, which was not detected in November.

**FIGURE 2:**
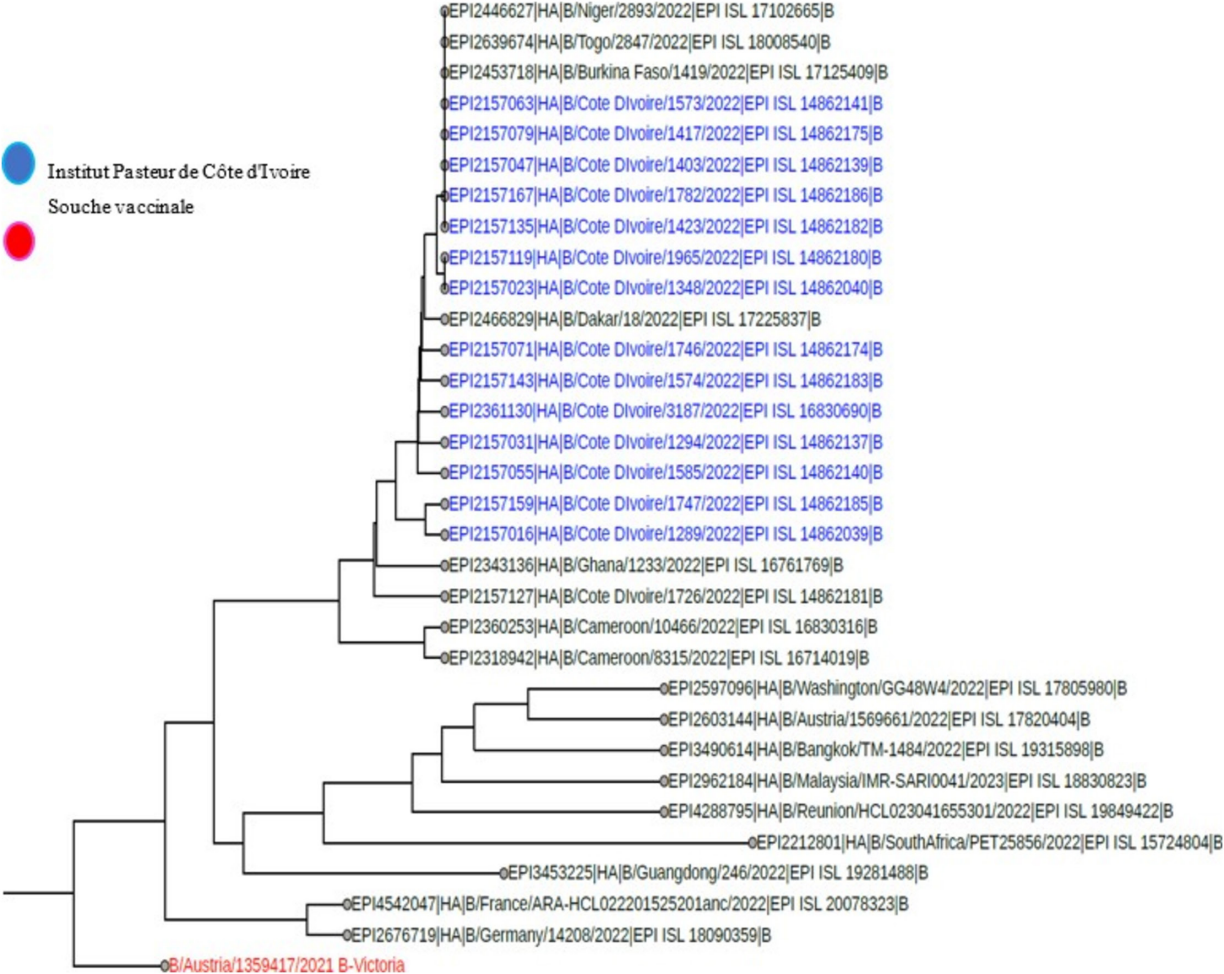
Phylogenetic comparison of influenza HA genes (B Victoria) Côte d'Ivoire, 2022.

**FIGURE 3:**
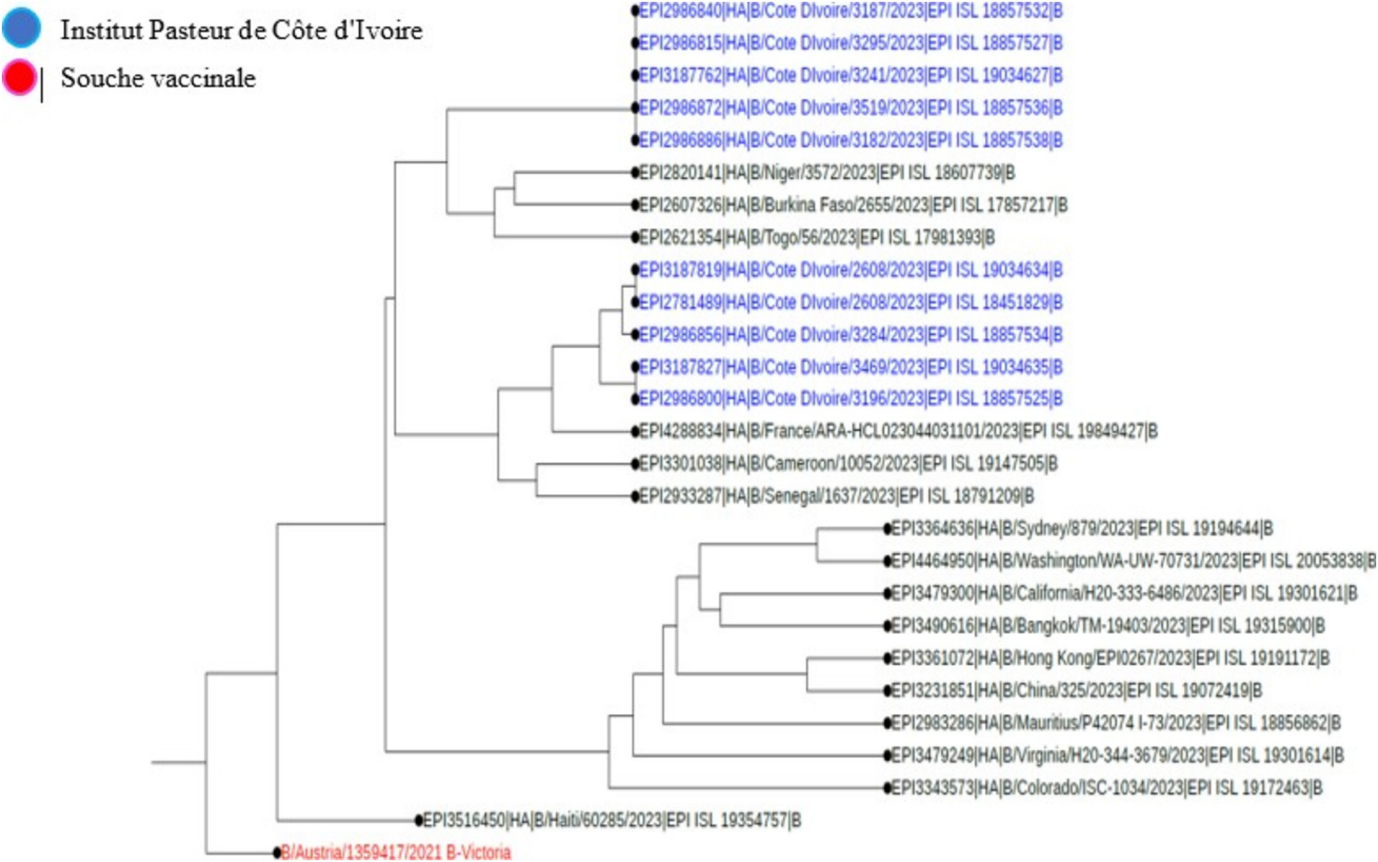
Phylogenetic comparison of influenza HA genes (B‐Victoria) Côte d'Ivoire, 2023.

**FIGURE 4:**
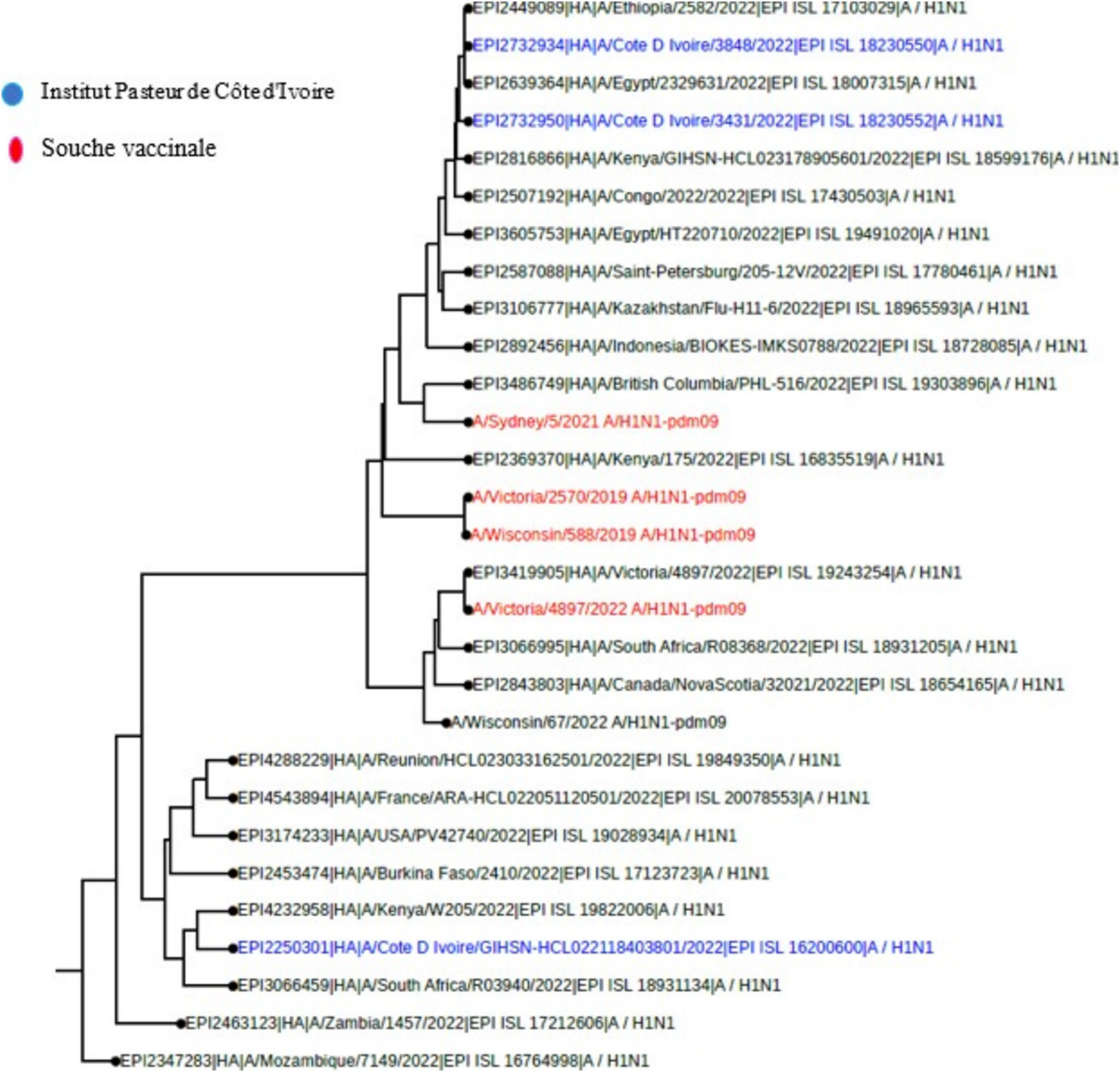
Phylogenetic comparison of influenza HA genes (H1N1) pdm09 Côte d'Ivoire, 2022.

**FIGURE 5:**
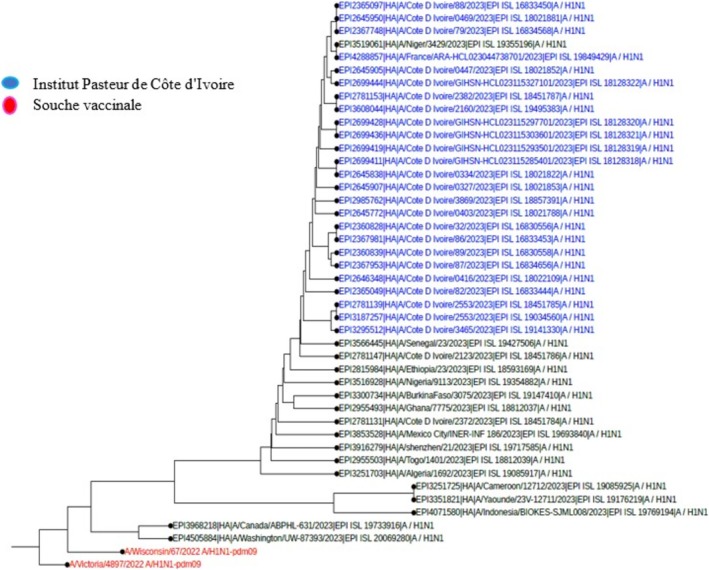
Phylogenetic comparison of influenza HA genes (H1N1) pdm09 Côte d'Ivoire, 2023.

**FIGURE 6:**
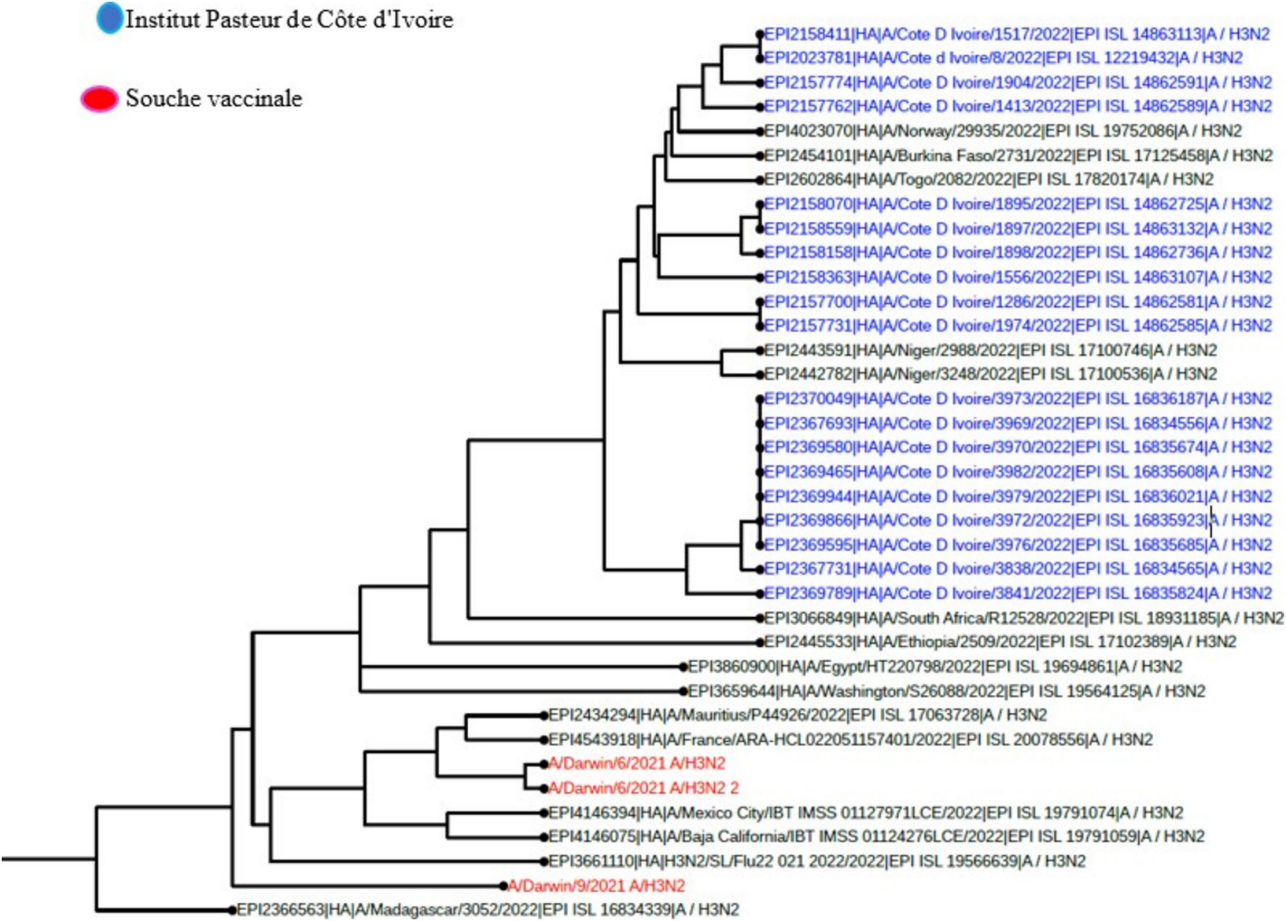
Phylogenetic comparison of influenza HA genes (H3N2) pdm09 Côte d'Ivoire, 2022.

**FIGURE 7:**
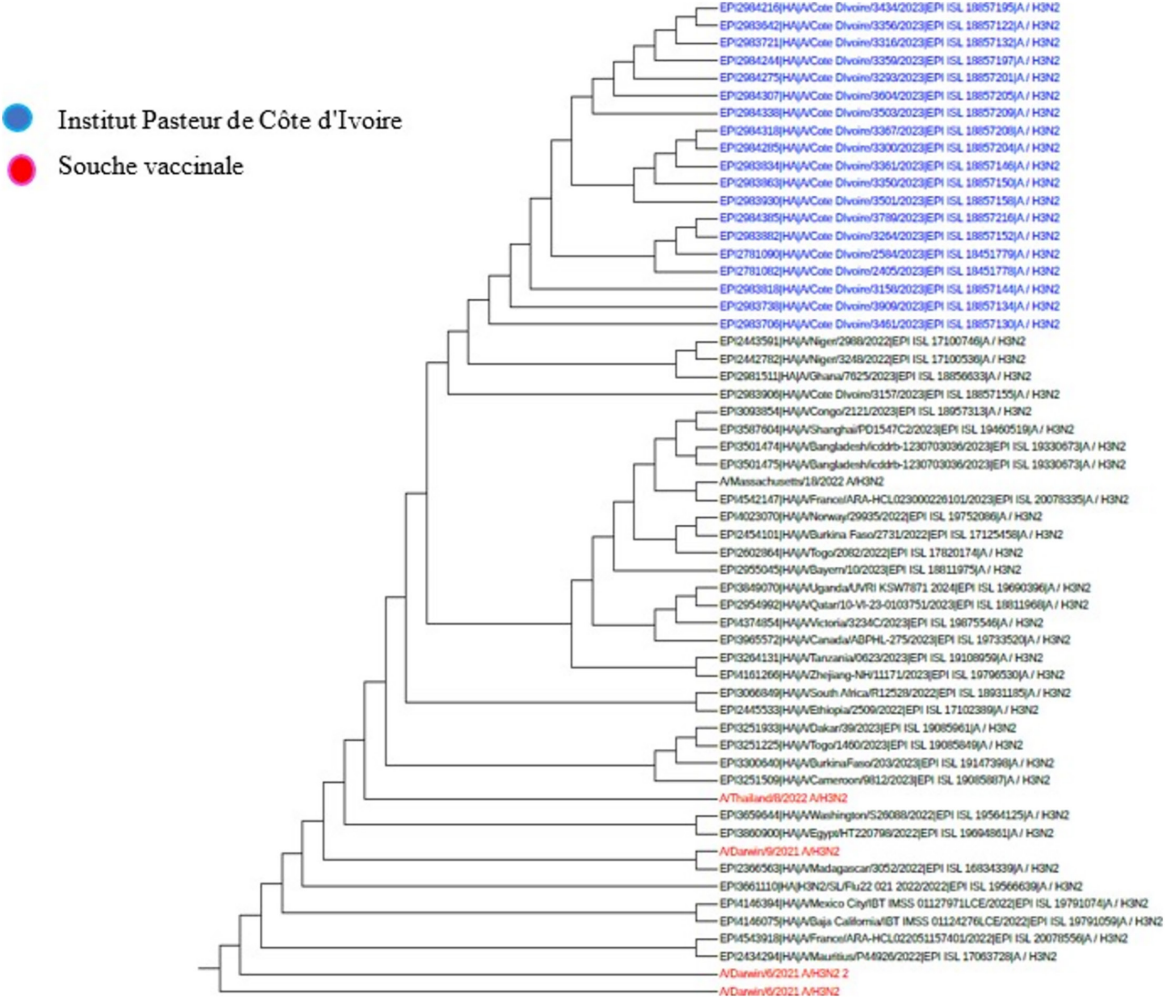
Phylogenetic comparison of influenza HA genes (H3N2) pdm09 Côte d'Ivoire, 2023.

Among the samples sent for influenza sequencing, 49 HA genes from the A(H3N2) virus, 57 HA genes from the A(H1N1) pdm09 virus, and 41 HA genes from the B Victoria lineage virus were successfully obtained. A phylogenetic analysis of the HA (hemagglutinin) gene sequences of influenza B viruses (Victoria lineage) detected in Côte d'Ivoire in 2022 and 2023 was performed to assess their genetic proximity to the recommended vaccine strain. The nucleotide sequences were aligned using MAFFT software, and the phylogenetic tree was constructed using the maximum likelihood (ML) method with IQ‐TREE software, with 1000 bootstrap replications to assess the robustness of the branches. As shown in Figures 2 and 3, the sequences obtained form a monophyletic cluster, reflecting local circulation of genetically related viruses, with all genetically characterized viruses belonging to the V1A.3a.2 genetic clade. These sequences show a high similarity to the B/Austria/1359417/2021‐like vaccine strain recommended by the WHO for the 2022 influenza season in the northern hemisphere. The HA gene sequences of B/Victoria influenza viruses detected in Côte d'Ivoire in 2023 revealed close genetic proximity to the vaccine strain recommended for the season. As shown in Figure 3, the local strains are mostly grouped in the same clade, with no major divergence from the reference vaccine strain. This homogeneity suggests good antigenic concordance between the circulating strains and the vaccine composition, which could promote optimal vaccine efficacy. The low genetic divergence observed suggests a good match between the circulating strains and the vaccine strain, which could indicate satisfactory vaccine efficacy during the study period. For A(H1N1) pdm09 viruses, phylogenetic analysis showed that they all belonged to genetic clade 6B.1A (Figures 4 and 5). The phylogenetic tree was constructed from the nucleotide sequences of the HA gene of the influenza A(H1N1) pdm09 virus detected in human cases in Côte d'Ivoire in 2022 and 2023. The local sequences are represented by clustered branches, indicating high genetic similarity and suggesting local circulation of closely related viruses. The reference vaccine strain is also included in order to compare its genetic proximity to the local strains. The analysis reveals a certain degree of conservation, but with genetic variations that may indicate partial antigenic drift. This comparison makes it possible to assess the suitability of the circulating strains and the vaccine strain for the purpose of monitoring and adapting vaccination recommendations. The tree was rooted and constructed using a Maximum Likelihood/Neighbor‐Joining algorithm. Phylogenetic analysis of the A(H3N2) virus indicates that all HA genes belonged to genetic clade 3C.2a.1b.2a.2a.3a (Figures 6 and 7).

**FIGURE 8:**
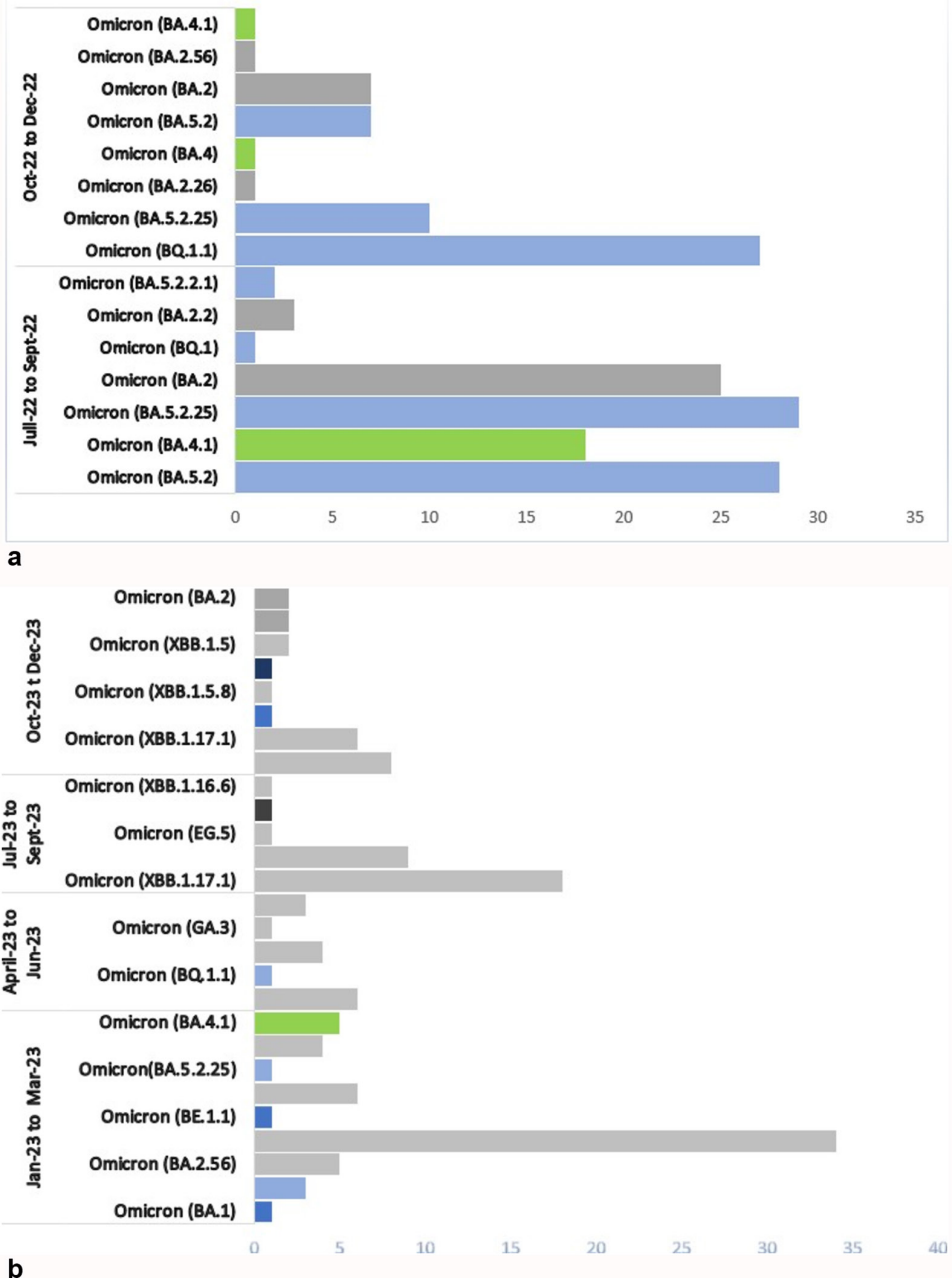
(a) Genetic diversity of circulating Omicron lineages and sublineages of SARS‐CoV‐2 in Côte d'Ivoire, 2022. (b) Genetic diversity of circulating Omicron lineages, sublineages and recombinants of SARS‐CoV‐2 in Côte d'Ivoire, 2023.

Regarding genomic surveillance of SARS‐CoV‐2 during the study period, 395 samples were subjected to whole‐genome sequencing using the MinION MK1C sequencer, following the standardized ARTIC SARS‐CoV‐2 consortium protocol. Among the 395 samples, 289 (73.16%) yielded high‐quality complete genomes, defined by ≥ 95% genome coverage and a sequencing depth of ≥ 30%. The validated sequences were submitted to the international GISAID database (https://www.gisaid.org/hcov19‐variants/). Phylogenetic analysis of the obtained sequences revealed the circulation of several Omicron lineages and sublineages. In 2022, three major lineages (BA.5, BA.4, and BA.2) comprising 12 [11] sublineages, were identified, the most frequent of which were BA.5.2.25 (17.26%), followed by BA.5.2 (16.66%), BQ1.1 (16.07%), and BA.4.1 (10.71%) (Figure 8a). In 2023, lineages BA.1, BA.2, BA.3 sublineages of BA.5, and recombinant variants were detected, with a predominance of the recombinant XBB.1.17.1 (39%) (Figure 8b). The temporal distribution of lineages revealed a decline in lineage diversity over the time with a predominance of recombinant virus.

**FIGURE 9:**
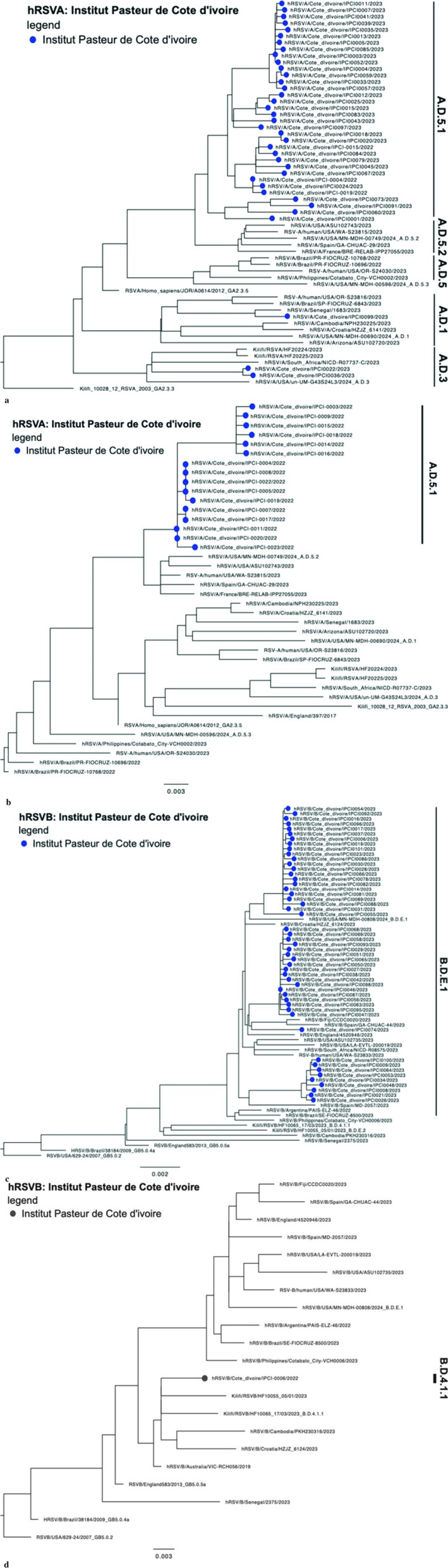
(a) Phylogenetic tree of WGS RSV A Côte d'Ivoire, 2022–2023. Phylogenetic analysis of the whole genome of RSV A showed that majority of the viruses from Côte d'Ivoire (blue circles) belonged to clade A.D.5.1, while two viruses belonged to A.D.3 and 1 A.D.1. (b) Phylogenetic tree of the G gene of RSV A Côte d'Ivoire, 2022. Phylogenetic analysis of the full G gene of RSV A showed that all 2022 viruses from Côte d'Ivoire (blue circles) belonged to clade A.D.5.1. (c) Phylogenetic tree of WGS RSV B Côte d'Ivoire, 2023. Phylogenetic analysis of the whole‐genome RSV B sequences showed that all 502,023 viruses from Côte d'Ivoire (blue circles) belonged to clade B.D.E.1. (d) Phylogenetic tree of the G gene of RSV B Côte d'Ivoire, 2022. Phylogenetic analysis of the full G gene of RSV B showed that the 2022 sample from Côte d'Ivoire (blue circle) belonged to clade B.D.4.1.1.

For RSV, of the 128 samples that were sequenced, 89 samples produced complete genomes, seven sample with full F and G sequences, 12 with full G sequences, and 16 samples had partial genomes with gaps in both F and G genes. Of note, only 1 whole genome sequence was obtained out of 18 RSV A viruses collected in 2022. Phylogenetic analysis of the whole genome of RSV A showed that majority of the viruses from Cote d'Ivoire (blue circles) belonged to clade A.D.5.1, while two viruses belonged to A.D.3 and 1 A.D.1 (Figure 9a). Phylogenetic analysis of the full G gene of RSV A showed that all 2022 viruses from Cote d'Ivoire (blue circles) belonged to clade A.D.5.1 (Figure 9b) Phylogenetic analysis of the whole‐genome RSV B sequences showed that all 2023 viruses from Cote d'Ivoire (blue circles) belonged to clade B.D.E.1 (Figure 9c). Phylogenetic analysis of the full G gene of RSV B showed that the 2022 sample from Cote d'Ivoire (blue circle) belonged to clade B.D.4.1.1 (Figure 9d).

## 4 Discussion

This study presents the findings of the first phase of the integration of SARS‐CoV‐2 and RSV surveillance into the existing influenza sentinel surveillance system in Côte d'Ivoire. Influenza virus surveillance has been operational in Côte d'Ivoire since 2006. The influenza reference laboratory was designated as the NIC as part of the WHO Global Influenza Surveillance and Response System (GISRS) in 2009. Since then, the surveillance system has contributed to improving knowledge of the epidemiology and seasonality of influenza viruses at the national level and supported global efforts in the selection of vaccine strains. Given the findings obtained with influenza sentinel surveillance, the expansion of the existing influenza sentinel surveillance system to include other major respiratory viruses, particularly RSV and SARS‐CoV‐2, appeared both timely and necessary to better understand the diversity and burden of viral respiratory infections circulating in the population. Both RSV and SARS‐CoV‐2 are responsible for significant morbidity and mortality worldwide, especially among vulnerable populations such as young children and the elderly. Integrating their surveillance within the influenza sentinel surveillance enhances early detection of co‐circulating respiratory pathogens, the country's preparedness and response capacities in the face of respiratory virus epidemics and pandemics, and, on the other hand, enables the optimization of resources.

The integration of RSV and SARS‐CoV‐2 into the sentinel influenza surveillance system in Côte d'Ivoire was implemented with a revision of the national protocol, in line with WHO recommendations [8, 15]. This revision focused on sentinel surveillance objectives, case definitions and reporting tools, to take into account the epidemiological and clinical specificities of each virus. One major adaptation concerned the case definition, to improve identification of RSV infections, particularly in young children and the elderly, by removing the requirement for fever as an inclusion criterion for patients to be recruited. Indeed, several studies indicate that a significant proportion of RSV infections in these groups may occur in the absence of fever [11, 16].

Similarly, although the revised protocol has incorporated specific symptoms such as anosmia and ageusia to improve detection of SARS‐CoV‐2 cases [17], none of the SARS‐CoV‐2 positive patients in our study reported these manifestations. This observation could reflect an evolution in the clinical picture of SARS‐CoV‐2, particularly with the predominance of Omicron sublineages during the study period, known to cause milder forms and less specific symptoms [18]. These findings suggest the need for ongoing evaluation of case definitions and recruitment practices in integrated surveillance systems to ensure their sensitivity, inclusiveness and ability to adapt to clinical and epidemiological developments.

During the study period, the sentinel surveillance system enabled the detection of cases of influenza viruses, RSV, and SARS‐CoV‐2. Of the 8316 samples tested, 12.55% were positive for at least one of these targeted viruses. RSV was the most frequently detected virus (5.63%), followed by influenza viruses (3.71%) and SARS‐CoV‐2 (3.22%). These findings indicate the interest of an integrated respiratory virus sentinel surveillance system in the simultaneous detection of various pathogens exhibiting similar clinical manifestations. The dominance of RSV we report is consistent with findings from other studies from sub‐Saharan African countries where RSV has been shown to be the leading cause of acute respiratory infection, particularly in young children [19]. Influenza viruses were more frequently detected among ILI cases than SARI cases. The positivity rate for influenza increased significantly between 2022 and 2023 in both ILI and SARI cases. These findings suggest a return to prepandemic levels of influenza virus circulation. During the COVID‐19 pandemic, the circulation of respiratory viruses decreased, largely due to the implementation of nonpharmaceutical interventions aimed at controlling the spread of SARS‐CoV‐2 [20]. In contrast, the detection of SARS‐CoV‐2 remained relatively stable among ILI cases over the two‐year period. However, among SARI cases, a decline in SARS‐CoV‐2 detection was observed. This trend may be attributed to increasing population‐level immunity, either through vaccination or prior infection, as well as a potential shift toward less severe clinical manifestations over time, particularly with the dominance of Omicron sublineages, which have shown reduced viral fitness [21]. Similar to prior findings, children younger than 2 years old were the most affected by RSV and influenza infections aligned with severe respiratory illnesses of viral origin, pointing out the susceptibility of this age group to ILI and SARI. Moreover, older adults with SARI also exhibited a significant statistical relationship between age and clinical severity of SARS‐CoV‐2 (*p* = 0.002), which demonstrates the need for adapting age‐specific risk assessment and indicates the need for emphasis on passive predication approaches for infants and active for the elderly. Regarding seasonality, each of the targeted viruses exhibited distinct temporal patterns, with partial overlap observed between influenza viruses and SARS‐CoV‐2. RSV showed significantly higher activity between May and October, peaking from June to September. This seasonal profile, with peaks occurring during both the rainy and dry seasons in Côte d'Ivoire, challenges the classical pattern of RSV seasonality typically associated with climatic conditions—particularly the rainy season—as described by previous studies [22, 23]. A number of studies carried out have highlighted a high degree of variability in RSV seasonality, characterized in some cases by dual seasonal peaks or prolonged circulation. These dynamics are not always clearly correlated with conventional meteorological parameters such as relative humidity or rainfall [24]. As for RSV, influenza viruses exhibited two peaks. The first peak is observed in February, coinciding with the dry season, while the second, more pronounced peak is observed in October, corresponding to the short rainy season. This seasonal pattern differs from that observed in temperate climate countries but remains similar to that observed in other regions of sub‐Saharan Africa, with a heterogeneous influenza seasonality less strictly associated with climatic parameters such as rainfall, temperature, or relative humidity [25]. Analysis of the monthly circulation of SARS‐CoV‐2 in Côte d'Ivoire between 2022 and 2023 reveals continuous transmission throughout the year, with two distinct peaks observed in February and September. The February peak coincided with an increase in influenza activity, which could suggest common transmission factors such as population mobility during the holiday season or climatic conditions. The second peak, observed in September, occurred in the absence of a concomitant influenza peak, highlighting the ability of SARS‐CoV‐2 to maintain sustained transmission outside the usual periods of seasonal respiratory virus circulation. It should be noted that the peaks were observed mainly during the dry seasons, which are characterized by high temperatures and low relative humidity. This seasonal profile observed over this period does not agree with the work of Koanda et al. [26], who highlighted a correlation between climatic parameters such as wind speed and temperature drop. Study protocols combining virological surveillance, ecological and meteorological data coupled with behavioral and socio‐epidemiological studies would be the best approach to elucidate this seasonality.

In terms of genomic surveillance, molecular characterization and phylogenetic analysis of the HA of influenza A(H3N2), A(H1N1) pdm09 and influenza B viruses in Côte d'Ivoire, highlighted the dominance of clades 3C.2 a.1b.2a.2a.3a for A(H3N2) viruses, 6B.1A.5a.2a for A(H1N1)pdm09 viruses and V1A.3a.2 for B/Victoria viruses. These data are in line with those observed in various regions of the globe, as reported by the WHO in its recommendations for influenza vaccine formulation [27, 28]. Phylogenetic analysis of HA genes from influenza B strains (Victoria lineage) detected in Côte d'Ivoire in 2022 and 2023 reveals a high degree of similarity with the B/Austria/1359417/2021‐like vaccine strain recommended by the WHO. The formation of a monophyletic cluster within the phylogenetic tree suggests local circulation of closely related strains, probably resulting from a single introduction or a few genetically similar introductions, followed by community transmission. The low genetic divergence observed between local strains and the vaccine strain supports the hypothesis of good antigenic match, which may have contributed to acceptable vaccine efficacy during the 2022 influenza season. These findings highlight the importance of continuous genomic surveillance to rapidly detect any antigenic drift that could compromise the efficacy of future vaccine formulations. Furthermore, these data confirm the relevance of the strain chosen for the region, while reinforcing the need for African countries to fully integrate viral genomics into sentinel surveillance systems. The rapid evolution of influenza viruses requires regular reassessment of the vaccine strains used, especially since B lineages are often neglected in sentinel surveillance analyses compared to A viruses. These data, collected at national level and shared via the WHO Collaborating Centers for Influenza or the GISAID platform, are essential for guiding the vaccine strain selection process each year. Genomic surveillance of SARS‐CoV‐2 in Côte d'Ivoire during the period 2022–2023 highlighted a high diversity of Omicron sublineages, notably BA.2.56 and BA.5.2.25 in 2022, followed by the predominance of recombinant lineages such as XBB.1.17.1 and GA.2 in 2023. However, from the second half of 2023 onward, a clear reduction in genetic diversity was observed, marked by the dominance of a few sublineages belonging predominantly to the XBB clade. This phenomenon was observed worldwide in the first half of 2023. The high immune pressure in the population, resulting from vaccination campaigns and previous infections, has probably favored the positive selection of more adapted variants, leading to the positive selection of a majority genotype [29]. Considerable efforts were made worldwide to strengthen sequencing of SARS‐CoV‐2 during the COVID‐19 pandemic, in particular after the identification of the critical role of certain variants, such as Alpha and Delta, in increasing the transmissibility and severity of the infection. This mobilization enabled real‐time monitoring of the viral evolution and contributed to the adaptation of public health measures. However, since the state of health emergency was lifted at, a gradual decline in genomic surveillance of SARS‐CoV‐2 has been observed worldwide, reflected by a marked drop in the number of sequences deposited in international databases such GISAID. This trend is particularly worrying in low‐income countries, where health priorities tend to be refocused on other pathologies perceived as more urgent. The continuation of genomic surveillance of SARS‐CoV‐2 in these contexts is all the more compromised as the infection is now predominantly benign, reducing the perception of its epidemiological importance and making it difficult to mobilize resources sustainably.

Phylogenetic analysis of RSV genes revealed that most RSV‐A viruses belonged to the A.D.5.1 sublineage. In contrast, RSV‐B viruses were predominantly from the B.D.E.1 clade. The A.D.5.1 sublineage was recently defined as part of the A.D.5 lineage in the updated phylogenetic classification proposed by Goya et al. in 2024 [30]. This sublineage has been detected in a limited number of countries, generally at low frequencies [31, 32]. Other genotypes identified in this study, including A.D.1, A.D.3, and A.D.5.2, have previously been reported in studies from China, the United States, and Europe. Most of the RSV‐B genomes sequenced in this study belonged to clade B.D.E.1. One virus from 2022 was classified as B.D.4.1.1. The dominance of B.D.E.1 has been documented in several recent studies [33, 34]. Since 2021, B.D.E.1 has become the globally dominant RSV‐B clade, gradually replacing B.D.4.1.1. This shift has been observed in countries such as the USA, Canada, and China. A study from the USA found that over 95% of RSV‐B strains belonged to the B.D.E.1 clade. A hypothesis that could explain the disappearance of the B.D.4.1.1 virus detected in 2022 is that this virus may have been introduced via travel or a localized event but failed to establish sustained transmission in the population. This hypothesis is supported by its absence in 2023.

In view of the forthcoming availability of a vaccine against RSV, several initiatives are currently being taken worldwide to strengthen RSV sentinel surveillance. However, in low‐income countries, many challenges remain, such as knowledge of the burden of RSV‐related morbidity, particularly in children under five, and data on the genetic diversity of circulating strains. This is partly due to the lack of systematic etiological diagnosis of acute respiratory infections in healthcare facilities, where virological detection capabilities are often inadequate or nonexistent. Strengthening epidemiological and virological surveillance of RSV in these settings, through surveillance networks such as GISRS, is therefore essential to guide vaccination strategies and assess their impact on transmission dynamics.

However, in practice, our sentinel surveillance system encountered difficulties in recruiting patients without fever. Over 94% of patients recruited had a fever. This suggests the existence of potential biases linked to the use of care and the habits of sentinel site agents, who would continue with influenza case definition. Fever is one of the main reasons for consultations, particularly in pediatric settings. In the absence of fever, many parents of children or the elderly resort to self‐medication or go directly to pharmacies to obtain medicines. These factors could limit the sensitivity of RSV sentinel surveillance and underline the need to reinforce the training and awareness of sentinel surveillance personnel to better capture acute respiratory infections occurring in the absence of fever.

## 5 Conclusion

This study demonstrates the feasibility of integrating RSV and SARS‐CoV‐2 sentinel surveillance into the existing influenza sentinel surveillance system in a resource‐limited setting. The implementation of this integration required a revision of the national influenza surveillance protocol. It enabled the collection of epidemiological and virological data, improving our understanding of the distribution of the targeted viruses within the general population. Age‐stratified analysis revealed distinct profiles: RSV infections were predominantly observed in children aged 0–2 years; SARS‐CoV‐2 infections affected mainly young children and individuals over 50 years of age, with a significant association between age and infection severity; influenza cases were primarily detected in children under 2 years and adults aged 25–49 years. The study also identified distinct, sometimes overlapping, seasonal patterns, as well as genetic characteristics consistent with global trends. However, to minimize inclusion bias, the integrated surveillance protocol must be adapted to the evolving epidemiological context of the viruses under surveillance and to specific objectives—particularly for RSV, where a better understanding of virus circulation in children under 2 years of age, the primary target group for upcoming vaccines, is essential. The sustainability and expansion of such an integrated surveillance system to other priority pathogens will not be possible without the mobilization of adequate resources. Integration of RSV and SARS‐CoV‐2 strengthened early detection, with summary of findings and future directions.

## Author Contributions

**Hervé A. Kadjo:** conceptualisation, methodology, writing – original draft, writing – review and editing. **Daouda Coulibaly:** conceptualisation, methodology, visualization. **Yakoura Karidja Ouattara:** data curation, formal analysis, software. **Sylla Aboubacar:** formal analysis. **Diané Maxime:** data curation, formal analysis. **Nguessan Konan:** investigation. **Kouakou Luc‐Venance:** investigation. **Kouassi Helene:** investigation. **Mboua Jean Marc:** investigation. **Kouakou Bertin:** data curation. **Adagba Marius:** data curation. **Edgard Adjogoua:** validation. **Ekra Daniel:** supervision. **Meite Syndou:** supervision.

## Ethics Statement

The sentinel surveillance of influenza and other respiratory viral infections is officially endorsed by the Ministry of Health of Côte d'Ivoire. This protocol is exempt from ethical considerations because it falls under public health surveillance. Written informed consent was obtained from all participants, and data were anonymized to ensure confidentiality.

## Conflicts of Interest

The authors declare no conflicts of interest.

## Data Availability

The data that supports the finding of this study are available in the national surveillance database. They can be made available upon request.
